# Antiresorptive treatment and talar collapse after displaced fractures of the talar neck: a long-term follow-up of 19 patients

**DOI:** 10.1080/17453674.2021.1915017

**Published:** 2021-04-18

**Authors:** Andreas Meunier, Lars Palm, Per Aspenberg, Jörg Schilcher

**Affiliations:** a Department of Orthopedics and Department of Biomedical and Clinical Sciences, Faculty of Health Science, Linköping University, Linköping;; b Wallenberg Centre for Molecular Medicine, Linköping University, Linköping, Sweden

## Abstract

Background and purpose — Displaced fractures of the talar neck are associated with a high risk of structural collapse. In this observational analysis we hypothesized that pharmacological inhibition of osteoclast function might reduce the risk of structural collapse through a reduction in bone resorption during revascularization of the injured bone.

Patients and methods — Between 2002 and 2014 we treated 19 patients with displaced fractures of the talar neck with open reduction and internal fixation. Of these, 16 patients were available for final follow-up between January and November 2017 (median 12 years, IQR 7–13). Among these, 6 patients with Hawkins type 3 fractures and 2 patients with Hawkins type 2b fractures received postoperative antiresorptive treatment (7 alendronate, 1 denosumab) for 6 to 12 months. The remaining 8 patients received no antiresorptive treatment. The self-reported foot and ankle score (SEFAS) was available in all patients and 15 patients had undergone computed tomography (CT) at final follow-up, which allowed evaluation of structural collapse of the talar dome and signs of post-traumatic osteoarthritis.

Results — The risk for partial collapse of the talar dome was equal in the 2 groups (3 in each group) and post-traumatic arthritis was observed in all patients. The SEFAS in patients with antiresorptive treatment was lower, at 21 points (95% CI 15–26), compared with those without treatment, 29 points (CI 22–35).

Interpretation — Following a displaced fracture of the talar neck, we found no effect of antiresorptive therapy on the rate of talar collapse, post-traumatic osteoarthritis, and patient-reported outcomes.

Talar neck fractures are often the result of a high-energy trauma and are associated with a high risk of complications (Dodd and Lefaivre 2015). Given the intraarticular location of the talus and its extensive cartilage coverage, its blood supply is vulnerable. Traumatic disruption of the blood supply during fracture of the talar neck leaves the talar dome at a high risk of avascular necrosis (AVN). The risk of AVN seems to be dependent on the presence of subtalar joint dislocation. If the joint is dislocated (type 2b and more severe types) the risk of AVN is 25–50%. No AVN is seen in cases of minor joint displacement (type 2a) (Vallier et al. 2014).

Interruption of the blood supply to the talar dome impairs the fracture healing capacity and bone remodeling. Subsequently, with revascularization of avascular areas, damaged osteocytes trigger the formation of osteoclasts (Glimcher and Kenzora 1979). These osteoclasts resorb the dead bone, and osteoblasts deposit new bone material (Hofstaetter et al. 2006). If the rate of bone resorption exceeds the rate of new bone formation the mechanical properties of the talar dome will deteriorate and may not be sufficient to withstand the forces transmitted during weight-bearing. The resulting collapse of the talus is associated with severe loss of function, limited range of motion and pain (Annappa et al. 2015).

Bisphosphonates reduce osteoclast function and thereby inhibit bone resorption. These agents have been proven to be successful in the treatment of several bone metabolic disorders, such as osteoporosis, osteogenesis imperfecta, Paget’s disease of bone, and metastatic bone disease. In addition, in the treatment of AVN of the femoral head, bisphosphonate treatment is associated with a reduction in the rate of structural collapse (Luo et al. 2014).

Considering the detrimental effects of post-traumatic AVN of the talus, and the low-risk profile of short-term antiresorptive treatment after a fracture (Abrahamsen 2010, Li et al. 2015), our department has encouraged postoperative treatment with antiresorptives after displaced fractures of the talar neck. In this retrospective analysis we evaluated the effects of this recommended treatment on the risk of talar collapse, the development of post-traumatic osteoarthritis (OA), and patient-reported outcome.

## Patients and methods

### Patients

**Figure 1.**
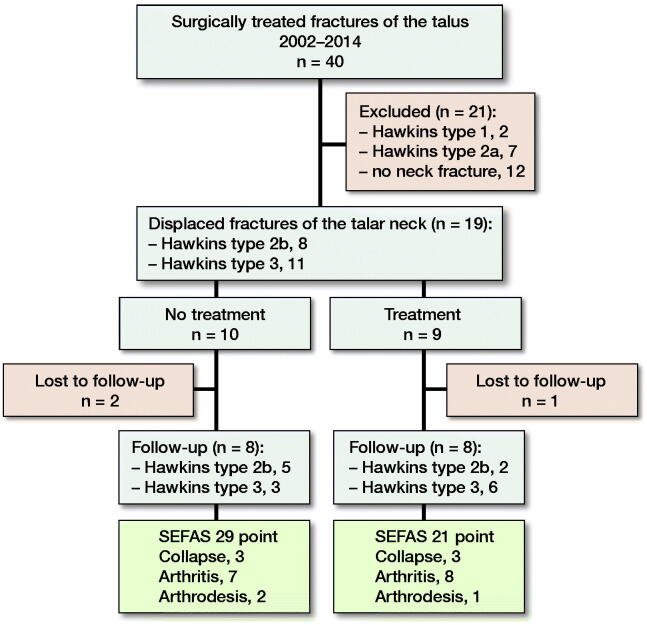
Flowchart of patient selection, treatment, and follow-up.

This is an observational analysis of patients who were treated surgically for fractures of the talus at Linköping University Hospital in the period 2002–2014 (Figure 1). During this period, we treated 40 consecutive patients with fracture of the talus with internal fixation. Individual radiographs taken pre- and postoperatively were collected and archived in our institutional Picture Archiving and Communication System.

### Fracture classification

**Figure 2.**
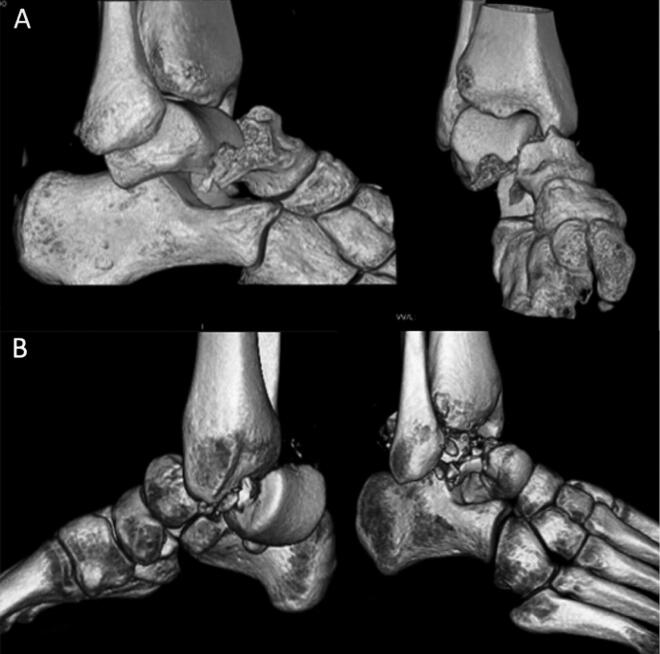
3-D CT reconstruction of fracture types represented in the study population: Hawkins type 2b (A) and type 3 (B).

Initial CT scans were used to classify fractures according to the modified Hawkins Classification (Vallier et al. 2014) (AM). Of the 40 patients in the register, 12 patients with fracture of the lateral process of the talus or without fracture of the talar neck were excluded. The remaining 28 patients were independently classified by 2 reviewers (AM and LP) who were blinded to treatment with antiresorptive drugs. The 2 reviewers showed excellent agreement for fracture types 1–3 in 26 cases. The remaining 2 patients were classified after consensus agreement. The final classification yielded patients with fractures of: Hawkins type 1 (n = 2); type 2a (displaced, but not dislocated subtalar joint) (n = 7) type 2b (dislocation of the subtalar joint but no tibio-talar dislocation; Figure 2a) (n = 8); and type 3 (both dislocation of the subtalar and the tibio-talar joint; Figure 2b) (n = 11). No type 4 fracture was identified. The numbers of patients with associated fractures of the talar dome were: 3 in the treatment group; and 2 in the group without antiresorptive treatment. The 19 patients with type 2b (n = 8) and type 3 (n = 11) fractures were called for final follow-up between January and November 2017.

Of the 19 patients who were called for follow-up, 16 patients (10 men) completed follow-up at a median of 12 years (IQR 7–13). Of these, 1 patient abstained from radiographic follow-up. The median age of the 16 patients in the study was 36 years (IQR 25–49). The patients’ background characteristics are presented in Tables 1 and 2.

**Table 1.** Patients’ characteristics and patient-reported outcomes using SEFAS and radiographic evaluation (CT) at final follow-up

Age	Sex	Type^a^	Associated injuries same foot	Trauma mechanism	Antiresorptive treatment ^b^	Follow-up (years)	Radiological findings	SEFAS score
31	Male	3	Ankle fracture	MVA	A, 6 months	12.7	Osteophytes	26
26	Male	3	Talus body	Fall > 2 m	A, 6 months	12.7	Partial collapse, osteophytes	14
46	Female	3	Talus body Ankle fracture	Fall > 2 m	A, 6 months	2.4	Partial collapse, osteophytes, JSN	24
61	Male	3	Ankle fracture	Crush injury	A, 6 months	5.8	Collapse with sequestration JSN	12
45	Male	3	Open talus body fracture	Fall **^c^**	A, 12 months	7.3	JSN, arthrodesis tibia-talus	32
38	Female	2b	Talus body, cuneiform, cuboid, navicular, and ankle fracture	MVA	A, 6 months	11.9	JSN	11
49	Female	2b	None	MVA	A, 12 months	4.5	JSN	17
22	Male	3	Ankle fracture	MVA	Denosumab	2.3	Osteophytes	31
34	Male	3	Ankle fracture	MVA	No	11.3	Osteophytes	24
55	Female	3	None	Low energy	No	12.4	JSN, arthrodesis talus-calcaneus	17
66	Male	3	Ankle fracture	Fall > 2 m	No	14.4	Collapse, arthrodesis, tibia-talus-calcaneus	36
19	Male	2b	None	MVA	No	11.4	Osteophytes	38
60	Male	2b	Talar body fracture	Low energy	No	11.8	Partial collapse, osteophytes	24
25	Female	2b	None	Fall > 2 m	No	12.7	Osteophytes	30
15	Female	2b	Talus body	MVA	No	13.4	Noradiographs available	43
21	Male	2b	Open distal tibia, calcaneus, and navicular fracture	MVA	No	9.6	Collapse, osteophytes	16
37	Male	2b	None	Low energy	No	No follow-up		
28	Male	3	None	MVA	No	No follow-up		
22	Male	3	Ankle fracture	MVA	A	No follow-up		

a Based on the modified Hawkins classification.

b A = alendronate

MVA = motor vehicle accident; JSN = joint space narrowing.

**Table 2.** Outcomes for the cohort as a whole and for the two groups.

Factor	n	Collapse	SEFAS mean (SD)	Mean age	Male sex
Overall	16	6	25 (9.7)	38	10
Treatment	8	3	21 (8.5)	40	5
Type 2b	2	0	14 (4.2)	44	0
Type 3	6	3	23 (8.4)	39	5
No treatment	8	3	29 (9.9)	37	5
Type 2b	5	2	30 (10.8)	28	3
Type 3	3	1	26 (9.6)	52	2

### Treatment

Surgical fixation was performed with either 1 or 2 screws inserted posterior (n = 12) or anterior (n = 7). Plate osteosynthesis was not used. Postoperatively, the patients were immobilized with a below-knee cast without weight-bearing for 6 to 8 weeks.

Based on the preference of the responsible surgical team, a treatment protocol with oral alendronate at 70 mg weekly was initiated within 14 days of the surgery and continued for 6 to 12 months (Table 1). At the end of the study period, the treatment protocol was switched to a single dose of 60 mg denosumab administered intravenously. Of the 19 patients with type 2b and type 3 fractures, 8 patients received alendronate and 1 received denosumab. The remaining 10 patients did not receive any antiresorptive treatment with either alendronate or denosumab (see Figure 1).

The prescription of antiresorptive drugs and the duration of treatment were obtained through a review of the medical charts and registered individual drug prescriptions. These pieces of information were obtained from the archived medical charts of patients treated between 2002 and 2008, and from the digitized medical charts and prescription system from 2008 and thereafter.

For evaluation of the outcome at the final follow-up (16 available patients) the patients were divided into 2 groups. The first group comprised those who received postoperative antiresorptive treatment (n = 8) and the second group contained those who did not receive such treatment (n = 8) (see Figure 1).

### Outcome measures

**Figure 3.**
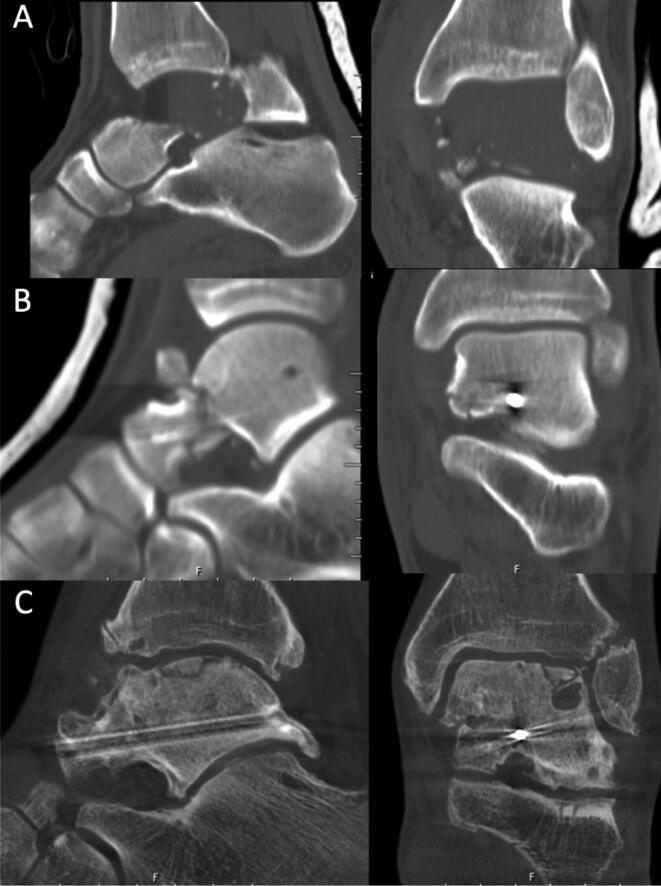
CT of a Hawkins type 3 fracture (A) in a 26-year-old man after a fall from a ladder. The fracture was treated with screw fixation (B), a non-weight-bearing cast for 8 weeks, and alendronate for 6 months. After 12.5 years, the fracture appeared to be healed on CT, showing post-traumatic talocrural OA and partial collapse of the talar dome (C).

**Figure 4.**
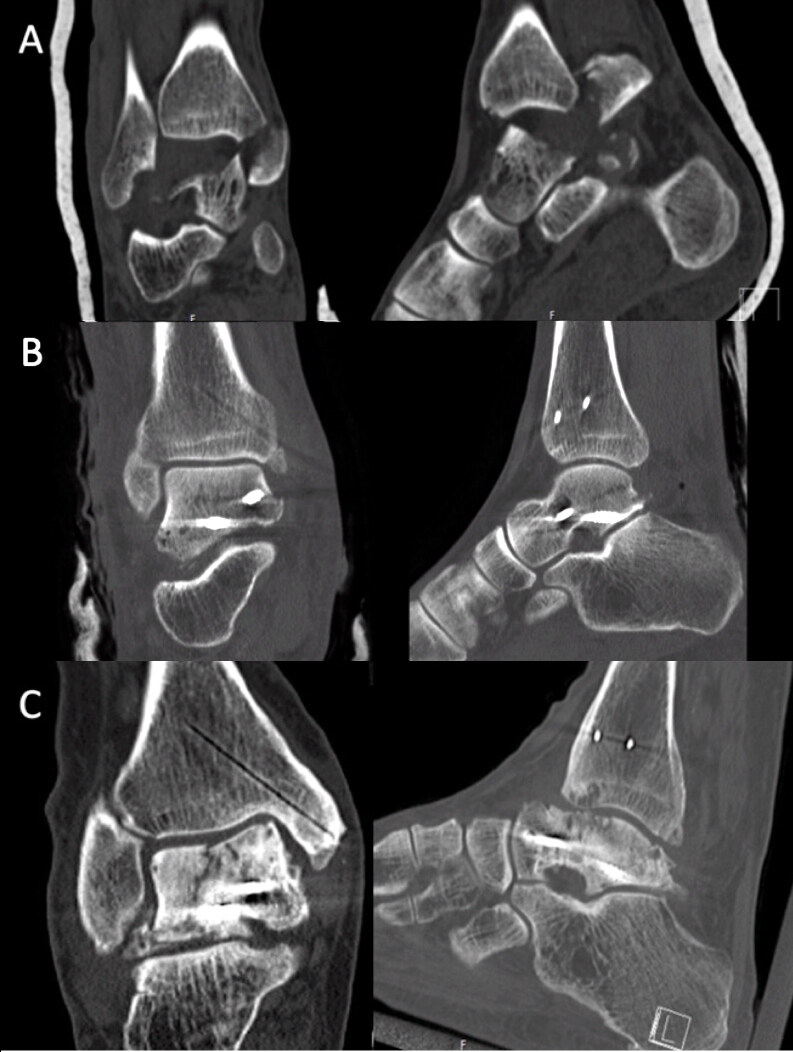
CT demonstrating a Hawkins type 3 fracture of the talus and associated medial malleolus fracture (A) in a 61-year-old man after a crush injury suffered during forest work. The fracture was treated with screw fixation (B), non-weight-bearing cast immobilization, and alendronate for 6 months. At 5.8 years after the surgery, the fracture had healed and the subchondral bone in the talar dome showed fragmentation due to collapse (C). Despite having poor ankle function (SEFAS of 12), the patient declined further surgery.

**Figure 5.**
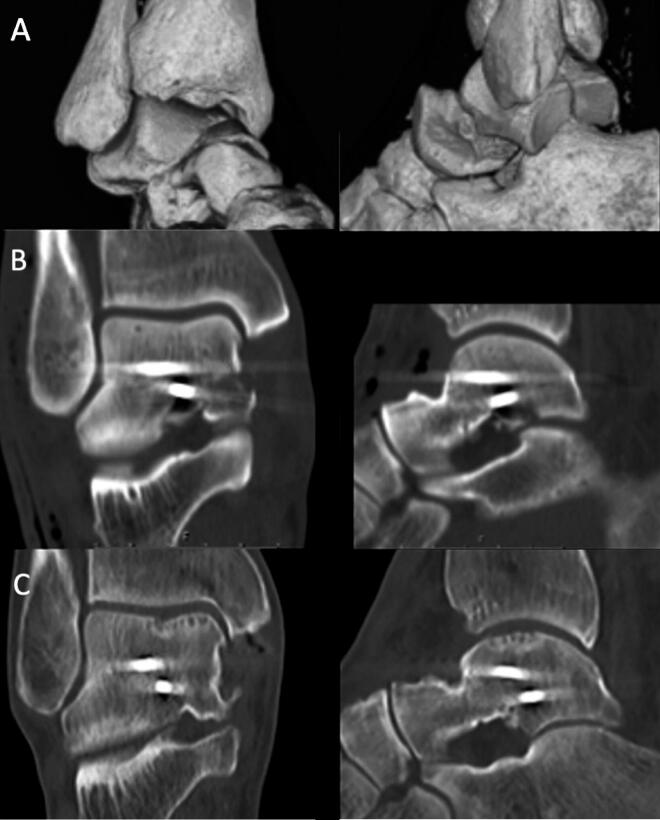
CT reconstruction demonstrating a Hawkins type 2b fracture with comminution of the talar dome (A) in a 60-year-old man after a low-energy fall. The fracture was reduced and fixed with 2 screws (B), followed by 6 weeks in a non-weight-bearing cast. No antiresorptive treatment was given. At 11.8 years postoperatively, the fracture was healed and the talar dome showed partial collapse (C). (SEFAS of 24).

The follow-up CT images were compared with the postoperative CT images in all planes to determine the degree of collapse (partial or total flattening or sequestration; Figures 3–5) of the talar dome. Osteoarthritis (OA) was defined as the occurrence of osteophytes on the joint surfaces or joint space narrowing of the talus or tibia (tibiotalar OA) or talus and calcaneus/navicular bone (subtalar OA).

The SEFAS system (Cöster et al. 2012) was used to evaluate patient-reported outcomes at the final follow-up. SEFAS is the validated Swedish modified translation of the New Zealand ankle questionnaire (Hosman et al. 2007).

### Statistics

We attempted to calculate the power of our sample to reject the null hypothesis. Due to the heterogeneity of the results obtained in previous studies and the small size of our cohort, we eventually refrained from a post-hoc power calculation. The SEFAS score is reported as the mean and standard deviation (SD) in accordance with previous publications (Cöster et al. 2017). Student’s t-test and relative risk (RR) with 95% confidence intervals (CI) were calculated to test differences in the outcome variables between groups.

### Ethics, funding, and potential conflicts of interest

The study was approved by the local Ethical Review Board (Dnr 2016/317-31), and all patients gave informed consent.

This study was supported by ALF-grants from Region Östergötland, Sweden. We thank the Knut and Alice Wallenberg Foundation for generous support. None of the authors has any conflict of interest.

## Results

In the radiographic follow-up, 6 (3 in each group) out of 15 patients (1 patient without treatment and a type 2b fracture had no radiographic follow-up) showed at least partial collapse of the talus, RR 0.9 (CI 0.4–2.0). For the 16 patients with available SEFAS scores at final follow-up, the mean score was 25 points (CI 20–30). For patients who received antiresorptive treatment, the mean SEFAS was 21 (CI 15–26), as compared with 29 points (CI 22–35) in the group without antiresorptive treatment. In all patients, post-traumatic OA was observed in at least 1 of the 3 possible joints. Arthrodesis was performed on 1 patient in the treatment group and on 2 patients in the untreated group (Figure 1 and Table 1).

## Discussion

In this observational analysis, we tested the hypothesis that pharmacologic inhibition of osteoclast function might reduce the risk of structural collapse after long-term follow-up. We found similar radiologic and patient-reported outcome in the 2 groups.

3 patients in each group showed at least partial collapse of the talar dome and all patients showed post-traumatic OA in at least 1 of the 3 examined joints. The rate of talar collapse in our study (6/16) is roughly twice that seen in a recent retrospective study of patients with fracture types 2b and 3 (8/43) (Vallier et al. 2014). This large difference may be related to multiple factors. The follow-up period in our study was much longer (median of 12 years compared with a mean of 2.5 years), and the sensitivity to detect areas of structural collapse using CT scans in our study is likely to be higher than that of the plain radiographs used in the Vallier study. Most previous studies did not evaluate collapse but looked at AVN only as defined by Hawkins, which makes comparisons with our study difficult. Moreover, the methods used for evaluating AVN have ranged from plain radiographs to MRI, and the definition of collapse differs across studies and among examiners (Dodd and Lefaivre 2015). In contrast to our hypothesis, the antiresorptive treatment administered in our study does not seem to affect the overall rate of structural collapse.

We found a 100% risk of post-traumatic OA in at least 1 of the examined joints (tibiotalar joint, subtalar joint, talonavicular joint). In this respect, there was no statistically significant difference between the 2 groups. This overall rate of OA is higher compared with those reported previously: 36/66 in the Vallier study (Vallier et al. 2014) and 68% in a systematic review (Halvorson et al. 2013). However, it is similar to the OA rate reported in a recent meta-analysis, in which 81% of patients showed signs of subtalar joint OA after more than 2 years of follow-up. In that study, talocrural OA was not included, and the authors noted that the majority of the included studies used only plain radiographs for the evaluation of OA. The high rate of post-traumatic OA we observed may be attributable to the long-term follow-up and thorough evaluation using CT.

Considering the high number of patients who suffered talar collapse and OA, the self-reported poor outcomes of the patients on the SEFAS are not surprising.

We did not investigate the development of AVN in the early postoperative phase, and we administered treatment irrespective of whether changes indicative of AVN were present or absent. The rationale here was to administer treatment before any bone resorption could occur. However, given the situation of interrupted perfusion after trauma, it might be more reasonable to initiate treatment once signs of revascularization become visible (Young et al. 2012). At this stage, the drug could reach the damaged area when bone resorption peaks and, thus, could inhibit osteoclast activity more effectively. Such an approach would be similar to the situation of atraumatic AVN of the femoral head, for which several clinical follow-up studies have shown a protective effect on structural collapse of the femoral head (Lai et al. 2005, Nishii et al. 2006, Agarwala et al. 2009, Agarwala and Vijayvargiya 2019). However, randomized trials using bisphosphonates to prevent femoral head collapse did not find a decrease in the need for arthroplasty (Chen et al. 2012, Lee et al. 2015).

The main strength of our study is its focus on the most severe types of talar neck fractures that have a high risk of structural collapse. In addition, the long-term follow-up, the high rate of patient participation in follow-up, and the rigorous evaluation of post-traumatic changes using CT ensure that our results are highly relevant for the clinical setting. Our radiographic analysis is based on comparisons of postoperative and follow-up CT scans with a higher sensitivity to detect collapse and OA, as compared with plain radiographs.

There are several limitations of our study. Treatment was not randomized but only recommended. Only half of the patients who could have received treatment were treated. Therefore, patients who received treatment may have been selected by surgeons with a higher level of experience and awareness of complications related to these injuries, which could have led to assignment of the worst cases to the treatment group. The treatment dosage and duration were empirical, based on experience gained from previous research. Patient compliance with the treatment regimen was questioned but was not registered in a logbook. Furthermore, our results might be biased by confounding elements. Because of the small sample size, we were not able to correct for potential confounders in this heterogeneous patient population.

In conclusion, for patients with displaced fractures of the talar neck, we found no effect of antiresorptive therapy on the rate of talar collapse, post-traumatic osteoarthritis, and patient-reported outcomes. However, because of the retrospective nature of the study and wide confidence intervals of our risk estimates, these results do not provide final evidence.

## References

[CIT0001] Abrahamsen B. Adverse effects of bisphosphonates. Calcif Tissue Int 2010; 86(6): 421–35. 20407762 10.1007/s00223-010-9364-1

[CIT0002] Agarwala S, Vijayvargiya M. Bisphosphonate combination therapy for non-femoral avascular necrosis. J Orthop Surg Res 2019; 14(1): 112. 31018848 10.1186/s13018-019-1152-7 PMC6480654

[CIT0003] Agarwala S, Shah S, Joshi V R. The use of alendronate in the treatment of avascular necrosis of the femoral head. J Bone Joint Surg Br 2009; 91-B(8): 1013–8. 10.1302/0301-620X.91B8.21518 19651826

[CIT0004] Annappa R, Jhamaria N L, Dinesh K V, Devkant, Ramesh R H, Suresh P K. Functional and radiological outcomes of operative management of displaced talar neck fractures. Foot (Edinb) 2015; 25(3): 127–30. 26025646 10.1016/j.foot.2015.03.004

[CIT0005] Chen C H, Chang J K, Lai K A, Hou S M, Chang C H, Wang G J. Alendronate in the prevention of collapse of the femoral head in nontraumatic osteonecrosis: a two-year multicenter, prospective, randomized, double-blind, placebo-controlled study. Arthritis Rheum 2012; 64(5): 1572–8. 22127729 10.1002/art.33498

[CIT0006] Cöster M, Karlsson M K, Nilsson J A, Carlsson A. Validity, reliability, and responsiveness of a self-reported foot and ankle score (SEFAS). Acta Orthop 2012; 83(2): 197–203. 22313352 10.3109/17453674.2012.657579 PMC3339537

[CIT0007] Cöster M C, Nilsdotter A, Brudin L, Bremander A. Minimally important change, measurement error, and responsiveness for the Self-Reported Foot and Ankle Score. Acta Orthop 2017; 88(3): 300–4. 28464751 10.1080/17453674.2017.1293445 PMC5434599

[CIT0008] Dodd A, Lefaivre K A. Outcomes of talar neck fractures: a systematic review and meta-analysis. J Orthop Trauma 2015; 29(5): 210–5. 25635362 10.1097/BOT.0000000000000297

[CIT0009] Glimcher M J, Kenzora J E. The biology of osteonecrosis of the human femoral head and its clinical implications, II: The pathological changes in the femoral head as an organ and in the hip joint. Clin Orthop Relat Res 1979; (139): 283–312. 455846

[CIT0010] Halvorson J J, Winter S B, Teasdall R D, Scott A T. Talar neck fractures: a systematic review of the literature. J Foot Ankle Surg 2013; 52(1): 56–61. 23153783 10.1053/j.jfas.2012.10.008

[CIT0011] Hofstaetter J G, Wang J, Yan J, Glimcher M J. Changes in bone microarchitecture and bone mineral density following experimental osteonecrosis of the hip in rabbits. Cells Tissues Organs 2006; 184(3-4): 138–47. 17409739 10.1159/000099620

[CIT0012] Hosman A H, Mason R B, Hobbs T, Rothwell A G. A New Zealand national joint registry review of 202 total ankle replacements followed for up to 6 years. Acta Orthop 2007; 78(5): 584–91. 17966016 10.1080/17453670710014266

[CIT0013] Lai K-A, Shen W-J, Yang C-Y, Shao C-J, Hsu J-T, Lin R-M. The use of alendronate to prevent early collapse of the femoral head in patients with nontraumatic osteonecrosis: a randomized clinical study. J Bone Joint Surg 2005; 87(10): 2155–9. 16203877 10.2106/JBJS.D.02959

[CIT0014] Lee Y K, Ha Y C, Cho Y J, Suh K T, Kim S Y, Won Y Y, Min B W, Yoon T R, Kim H J, Koo K H. Does zoledronate prevent femoral head collapse from osteonecrosis? A prospective, randomized, open-label, multicenter study. J Bone Joint Surg Am 2015; 97(14): 1142–8. 26178889 10.2106/JBJS.N.01157

[CIT0015] Li Y T, Cai H F, Zhang Z L. Timing of the initiation of bisphosphonates after surgery for fracture healing: a systematic review and meta-analysis of randomized controlled trials. Osteoporos Int 2015; 26(2): 431–41. 25266485 10.1007/s00198-014-2903-2

[CIT0016] Luo R B, Lin T, Zhong H M, Yan S G, Wang J A. Evidence for using alendronate to treat adult avascular necrosis of the femoral head: a systematic review. Med Sci Monit 2014; 20: 2439–47. 25424061 10.12659/MSM.891123 PMC4257480

[CIT0017] Nishii T, Sugano N, Miki H, Hashimoto J, Yoshikawa H. Does alendronate prevent collapse in osteonecrosis of the femoral head? Clin Orthop Relat Res 2006; 443: 273–9. 16462451 10.1097/01.blo.0000194078.32776.31

[CIT0018] Vallier H A, Reichard S G, Boyd A J, Moore T A. A new look at the Hawkins classification for talar neck fractures: which features of injury and treatment are predictive of osteonecrosis? J Bone Joint Surg Am 2014; 96(3): 192–7. 24500580 10.2106/JBJS.L.01680

[CIT0019] Young M L, Little D G, Kim H K. Evidence for using bisphosphonate to treat Legg-Calvé-Perthes disease. Clin Orthop Relat Res 2012; 470(9): 2462–75. 22270467 10.1007/s11999-011-2240-0 PMC3830104

